# Maintenance-energy requirements and robustness of *Saccharomyces cerevisiae* at aerobic near-zero specific growth rates

**DOI:** 10.1186/s12934-016-0501-z

**Published:** 2016-06-17

**Authors:** Tim Vos, Xavier D. V. Hakkaart, Erik A. F. de Hulster, Antonius J. A. van Maris, Jack T. Pronk, Pascale Daran-Lapujade

**Affiliations:** Department of Biotechnology, Delft University of Technology, Van der Maasweg 9, 2629 HZ Delft, The Netherlands

**Keywords:** Yeast, Retentostat, Zero growth, Robustness, Heat-shock, Aerobic, Energetics, Maintenance

## Abstract

**Background:**

*Saccharomyces cerevisiae* is an established microbial platform for production of native and non-native compounds. When product pathways compete with growth for precursors and energy, uncoupling of growth and product formation could increase product yields and decrease formation of biomass as a by-product. Studying non-growing, metabolically active yeast cultures is a first step towards developing *S. cerevisiae* as a robust, non-growing cell factory. Microbial physiology at near-zero growth rates can be studied in retentostats, which are continuous-cultivation systems with full biomass retention. Hitherto, retentostat studies on *S. cerevisiae* have focused on anaerobic conditions, which bear limited relevance for aerobic industrial processes. The present study uses aerobic, glucose-limited retentostats to explore the physiology of non-dividing, respiring *S. cerevisiae* cultures, with a focus on industrially relevant features.

**Results:**

Retentostat feeding regimes for smooth transition from exponential growth in glucose-limited chemostat cultures to near-zero growth rates were obtained by model-aided experimental design. During 20 days of retentostats cultivation, the specific growth rate gradually decreased from 0.025 h^−1^ to below 0.001 h^−1^, while culture viability remained above 80 %. The maintenance requirement for ATP (m_ATP_) was estimated at 0.63 ± 0.04 mmol ATP (g biomass)^−1^ h^−1^, which is ca. 35 % lower than previously estimated for anaerobic retentostats. Concomitant with decreasing growth rate in aerobic retentostats, transcriptional down-regulation of genes involved in biosynthesis and up-regulation of stress-responsive genes resembled transcriptional regulation patterns observed for anaerobic retentostats. The heat-shock tolerance in aerobic retentostats far exceeded previously reported levels in stationary-phase batch cultures. While in situ metabolic fluxes in retentostats were intentionally low due to extreme caloric restriction, off-line measurements revealed that cultures retained a high metabolic capacity.

**Conclusions:**

This study provides the most accurate estimation yet of the maintenance-energy coefficient in aerobic cultures of *S. cerevisiae*, which is a key parameter for modelling of industrial aerobic, glucose-limited fed-batch processes. The observed extreme heat-shock tolerance and high metabolic capacity at near-zero growth rates demonstrate the intrinsic potential of *S. cerevisiae* as a robust, non-dividing microbial cell factory for energy-intensive products.

**Electronic supplementary material:**

The online version of this article (doi:10.1186/s12934-016-0501-z) contains supplementary material, which is available to authorized users.

## Background

The yeast *Saccharomyces cerevisiae* is an established microbial host for the production of native yeast metabolites as well as non-native products [1]. Production of many of these compounds, including phenylpropanoids, isoprenoids, heterologous proteins and lipids [2–4] from glucose requires a net input of ATP. The maximum ATP yield from glucose is obtained when its dissimilation occurs exclusively via respiration. In *S. cerevisiae*, a completely respiratory sugar metabolism requires aerobic conditions and sugar-limited cultivation at low to intermediate specific growth rates [5]. In industry, these requirements are usually met by sugar-limited, aerobic fed-batch cultivation. Due to oxygen-transfer and cooling constraints, aerobic fed-batch processes typically involve low specific growth rates [6, 7]. However, biomass-specific production rates (q_p_) of products whose biosynthesis from sugar requires a net input of ATP typically show a positive correlation with specific growth rate [3, 4, 8, 9]. Understanding and, ultimately, breaking this correlation between growth and product formation by improving specific rates of product formation at low specific growth rates, is an important target for optimizing productivity and product yields in aerobic, sugar-limited fed-batch cultures.

**Fig. 1:**
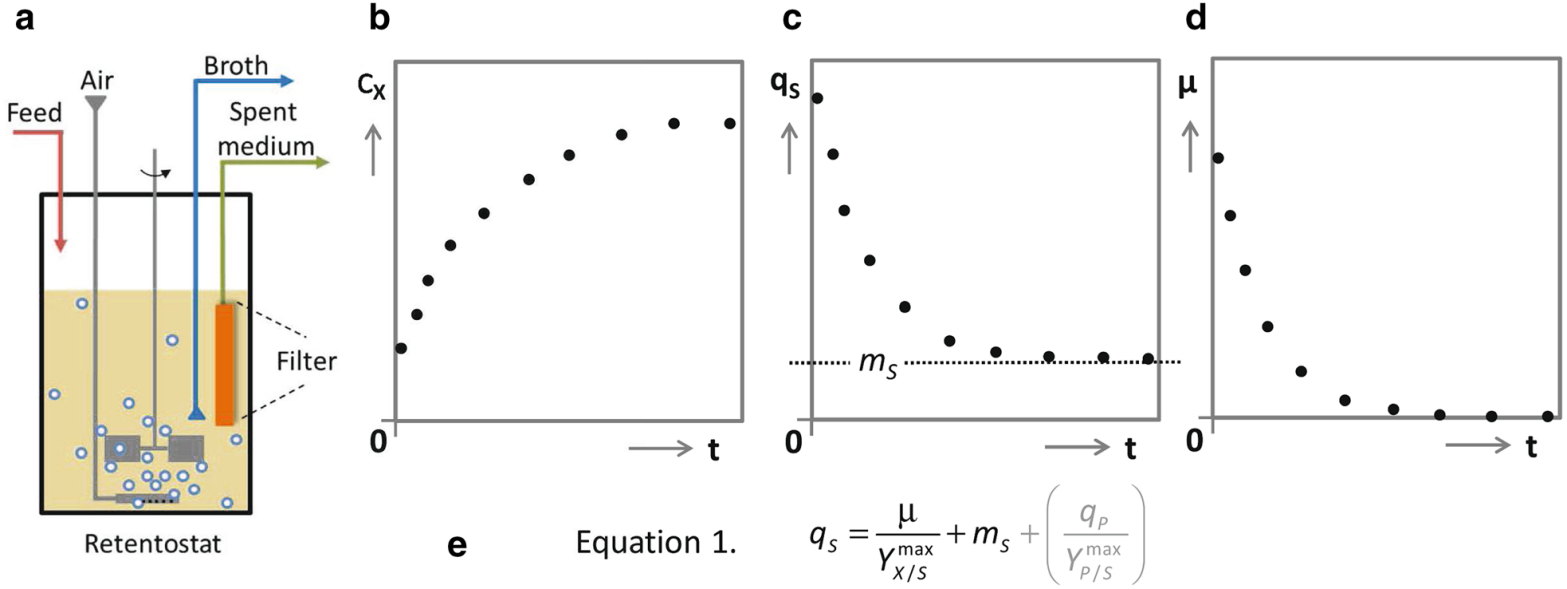
Schematic representation of retentostat set-up and simulated profiles of biomass accumulation (C_X_), glucose consumption rate (q_S_) and specific growth rate (µ) during prolonged retentostat cultivation. The retentostat is a continuous bioreactor system in which the outflow can be switched from whole-broth removal to complete cell retention through a filter probe (**a**). After switching from chemostat cultivation to retentostat mode, biomass accumulates in the bioreactor (**b**), which gradually decreases the glucose availability per unit of biomass. This decrease ultimately results (**c**) in specific glucose consumption rates that can only meet energy demands for cellular maintenance (m_S_), thereby causing near-zero specific growth rates (**d**). The distribution of the carbon and energy source over growth, maintenance and product formation (not indicated in the *plots*) is mathematically captured by an extended Pirt equation (**e**), in which Y_X/S_^max^ is the maximum theoretical biomass yield, q_P_ is the specific production rate of a product whose synthesis requires metabolic energy, and Y_P/S_^max^ is the maximum theoretical yield of this product on substrate

In addition to the relation between q_P_ and specific growth rate, microbial product formation at low specific growth rates is strongly influenced is by the metabolic-energy requirement of microorganisms for maintaining cellular integrity and viability. In a first analysis, this maintenance-energy requirement is often assumed to be growth-rate independent [3, 10]. Distribution of carbon- and energy substrate over growth and cellular maintenance can then be described by the Pirt equation [11], which can be modified to include ATP-requiring product formation (see equation in Fig. 1). The Pirt equation describes how the fraction of the energy substrate that needs to be dissimilated to fulfil maintenance energy requirements increases as the specific growth rate in, for example, an aerobic, sugar-limited fed-batch process decreases. In slow-growing aerobic industrial fed-batch processes this increasing impact of maintenance requirements has a major negative impact on product yields and productivities [3].

Analysis of the physiology of extremely slow growing yeast cultures can provide relevant, quantitative information on the maintenance-energy requirements of *S. cerevisiae* and for developing this yeast into a non-growing cell factory [12–16]. Retentostats are continuous cultivation devices with full biomass retention that have been designed to study microbial physiology at near-zero growth rates [17, 18]. Retentostat cultivation typically starts with a steady-state chemostat culture, operated at a low dilution rate. After reaching steady state, the chemostat culture is switched to retentostat mode by redirecting the effluent through a filter unit that ensures full biomass retention (Fig. 1). The constant, growth-limiting feed of glucose will then result in biomass accumulation (C_X_), while the amount of substrate available per cell per unit of time decreases over time (Fig. 1). This decreased substrate availability results in decreasing specific substrate consumption rates (q_S_) which, after prolonged retentostat cultivation, asymptotically approach the cellular energy-substrate requirement for maintenance (m_S_). Since, in this situation, no energy-substrate is available for growth, the specific growth rate (µ) asymptotically approaches zero (Fig. 1).

Retentostat cultures have mostly been used in the early 1990’s to investigate the response of prokaryotes to extreme energy limitation. At extremely low growth rates, many bacteria, including *Escherichia coli*, display an alarmone-mediated stringent response. This coordinated response enables cultures to more efficiently withstand nutrient scarcity by down regulation of energy-intensive cellular processes and, therefore, a reduction of the maintenance-energy requirement [19–21]. Retentostats have recently been used to study the physiology of *S. cerevisiae* at near-zero growth rates under anaerobic conditions [12–16]. Even at extremely low specific growth rates, the maintenance requirement of *S. cerevisiae* in these anaerobic chemostat cultures was shown to be growth-rate independent [12]. A decrease of the ATP-turnover of non-growing cultures was only observed when anaerobic, retentostat-grown *S. cerevisiae* cultures were switched to glucose starvation and energy metabolism became dependent on metabolism of storage carbohydrates [13]. Transcriptome responses during anaerobic retentostats encompassed many genes whose transcription was previously shown to be growth-rate correlated in faster growing cultures, as well as an increased expression of genes involved in resistance to a variety of stresses [14]. Consistent with the latter observation, yeast cells grown at low specific growth rates acquire a strongly increased robustness towards heat shock and an increased chronological life span [13, 22].

Since previous retentostat studies on *S. cerevisiae* were exclusively performed under anaerobic conditions, it remains unclear how oxygen availability affects its physiology at extremely low specific growth rates. Oxygen is known to have multiple effects on cellular biology. Even in *S. cerevisiae*, which has a rather low efficiency of oxidative phosphorylation, fully respiratory dissimilation of glucose yields eightfold more ATP than alcoholic fermentation, which is the sole dissimilatory pathway under anaerobic conditions [23]. This higher ATP yield supports higher biomass yields and, if the maintenance-requirement for ATP (m_ATP_) is the same in aerobic and anaerobic cultures, should lead to a lower m_S_ than observed in anaerobic cultures. Since biomass yield and maintenance-energy requirement affect the dynamics of retentostats, these differences should also be taken into account in the design of feed regimes that result in a smooth transition from exponential growth to near-zero growth rates. Despite the industrial relevance of maintenance-energy requirements, accurate experimental estimates of m_S_ and m_ATP_ for aerobic, sugar-limited cultures of *S. cerevisiae* on synthetic medium are not available. The assumption that m_ATP_ in aerobic cultures is the same as in anaerobic cultures [24], can result in over- or underestimation of the m_s_ of aerobic cultures. In anaerobic cultures, presence of the anaerobic growth factor oleic acid [25] and of ethanol and organic acids might increase m_ATP_. Similarly, detoxification of reactive oxygen species (ROS) and repair of ROS-induced damage may lead to increased maintenance energy requirements in aerobic cultures [26]. ROS, which can contribute to cellular aging, could also accelerate cell death of aerobic, non-dividing and chronologically aging yeast cultures [27]. A further question that remains to be addressed is whether and to what extent extremely slow-growing *S. cerevisiae* cultures retain a high metabolic capacity, which is a prerequisite for efficient product formation. Previous studies showed that glucose-limited aerobic cultures of *S. cerevisiae* retain a high capacity of glycolysis (the highway for sugar assimilation) at specific growth rates down to 0.05 h^−1^ [28], but no data are available on the glycolytic capacity at near-zero growth rates.

The goal of the present study is to quantitatively analyse maintenance-energy requirement, robustness and glycolytic capacity of *S. cerevisiae* in aerobic cultures grown at near-zero growth rate. To this end, regimes for aerobic retentostat cultivation were designed and implemented that enabled a smooth transition from exponential growth to near-zero growth rates. In addition to quantitative physiological analyses, transcriptome analysis was performed to investigate cellular responses to near-zero growth in aerobic cultures and to compare these with previously published transcriptome data obtained from anaerobic retentostats.

## Results

### Design of a regime for smooth transition to near-zero growth rates in aerobic retentostats

Growth rate dynamics and biomass accumulation in retentostat cultures mainly depend on two condition-dependent and strain-specific parameters: the theoretical maximal biomass yield (Y_X/S_^max^) and the maintenance coefficient (m_S_). To predict the impact of these parameters on growth dynamics in aerobic retentostat cultures, a model based on the Pirt definition of resource allocation (see Fig. 1) was used. Y_X/S_^max^ was estimated from published data on aerobic, glucose-limited chemostat cultures of the *S. cerevisiae* strain used in this study (0.5 g g^−1^ [28]). Since no accurate estimates for the aerobic m_S_ are available, model-based simulations were performed with a range of m_S_ values that were based on the m_S_ calculated from anaerobic retentostat experiments (biomass-specific glucose consumption for maintenance: 0.5 mmol g_X_^−1^ h^−1^, [12]) and assuming a P/O ratio of 1.0 for aerobic, respiring cultures of *S. cerevisiae* [23, 29], which leads to an eightfold higher ATP yield from respiratory sugar dissimilation than from alcoholic fermentation.

**Fig. 2:**
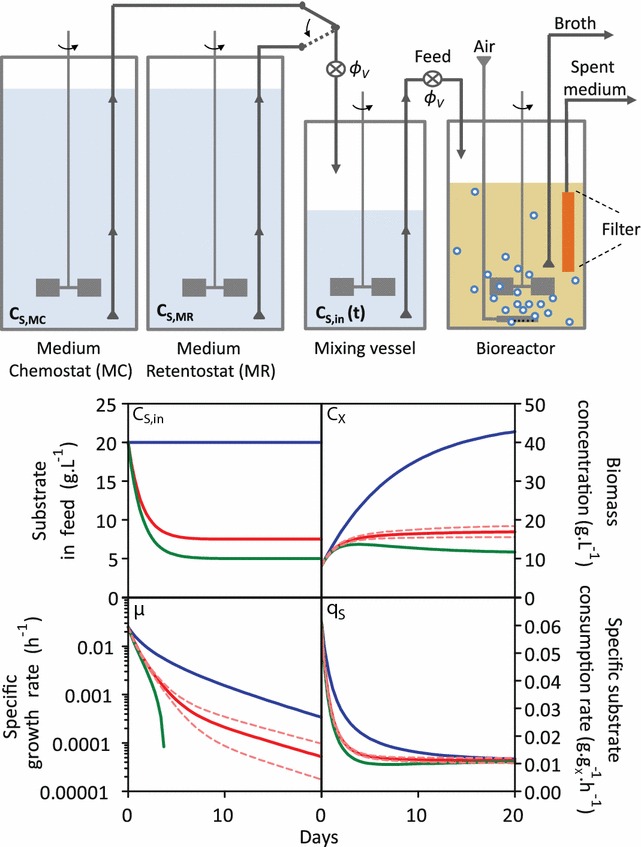
Setting up aerobic retentostat cultures. **a** Retentostat cultures (bioreactors) were started from a steady-state chemostat culture with an ingoing glucose concentration (C_S,MC_) of 20 g L^−1^. At the start of the retentostats (t = 0 h), the feed to the mixing vessel was switched to the medium reservoir for the retentostat cultivation (as indicated by the *arrow*). The process was modelled for three different concentrations of glucose in the medium reservoir for the retentostat cultures (C_S,MR_). **b** Profiles of biomass concentration (C_X_), specific glucose consumption rate (q_S_) and specific growth rate (µ) in time were predicted with a mathematical model, based on glucose concentration in the feed coming from the mixing vessel. *Blue lines* indicate a scenario in which C_S,MC_ = C_S,MR_ = 20 g L^−1^, *green lines* indicate C_S,MR_ = 5 g L^−1^, and* red lines* indicate C_S,MR_ = 7.5 g L^−1^. *Dotted lines* indicate simulations for which 10 % higher or lower maintenance values were considered in the model (see “Methods” section). The operational conditions applied in the experiments in this study correspond to the *red lines*

Initial model simulations were performed based on the assumption that no loss of viability occurs during retentostat cultivation and with the same feed regime that was previously used for anaerobic retentostats (constant dilution rate of 0.025 h^−1^ and a glucose concentration in the feed of 20 g L^−1^ [12]). This resulted in a predicted accumulation of biomass to a concentration of ca. 45 g L^−1^, Fig. 2, blue line), which was considered to present a substantial risk of clogging the filter unit. Moreover, in this simulation, near-zero growth rates (i.e. specific growth rates below 0.001 h^−1^) were only reached after multiple weeks of operation (Fig. 2, blue line), which was considered to be impracticable.

Near-zero growth rates can be reached faster by decreasing the glucose concentration in the medium for the retentostat culture (C_S,MR_) compared to the glucose concentration in the medium for the chemostat culture (C_S,MC_). However, care should be taken to avoid scenarios in which the glucose supply changes suddenly or transiently decreases below the culture’s maintenance-energy demand, which might affect cellular viability. Introduction of an additional medium mixing vessel (Fig. 2), allowed for a controlled, smooth transition of the ingoing glucose concentration (C_S,in_) into the retentostat culture, whilst maintaining a constant flow of medium (ɸ_V_) and thereby a constant dilution rate. To incorporate the mixing vessel the model was expanded with Eq. 2 and simulations were performed for experimental design of C_S,MR_ and the volume of the mixing vessel (V_S_ in liters) (Fig. 2).

2 $$\frac{{dC_{S,in} }}{dt} = \frac{{\phi_{V} }}{{V_{S} }}C_{S,MR} - \frac{{\phi_{V} }}{{V_{S} }}C_{S,in}$$

Figure 2 depicts the modelling output when C_S,MR_ equals C_S,MC_ (blue lines) and when C_S,MR_ was decreased to 7.5 or 5 g L^−1^ (solid red and green lines, respectively) assuming an m_S_ of 0.011 g g_X_^−1^ h^−1^.

In the simulations, values of C_S,MR_ of 5 g L^−1^ and lower resulted in ‘negative growth’, indicating that the model predicted glucose starvation and cell death. Since, in extremely slow growing cultures, glucose is predominantly used for maintenance, growth dynamics in retentostats are particularly sensitive to variations in m_S_. Accordingly, a 20 % change in m_S_ resulted in a fivefold difference in the predicted specific growth rates after 20 days of retentostat cultivation (dashed lines in Fig. 2). Based on these simulations, operational conditions were chosen such that the prediction complied to the following requirements: (i) near-zero growth rates (µ < 0.001 h^−1^) achieved within 2 weeks of retentostat cultivation, (ii) prevention of sudden changes in q_S_ and glucose starvation, (iii) the conditions led to a sizeable difference between initial and final biomass concentrations, (iv) previous criteria met for a range of m_S_ values, and (v) final biomass concentration kept below 30 g L^−1^ to prevent filter clogging (Fig. 2, red line). The chosen operational conditions are described in Fig. 2, and correspond to the red line.

### Maintenance-energy requirements in aerobic retentostat cultures

**Fig. 3:**
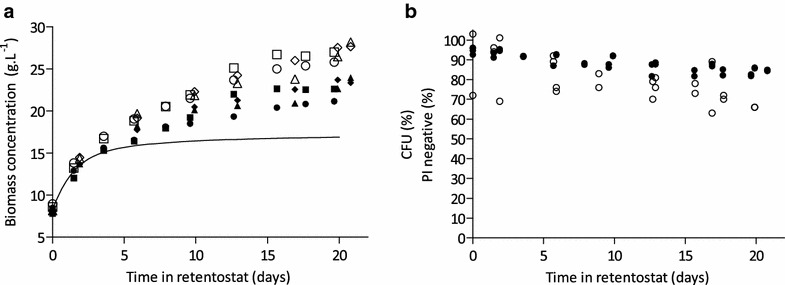
Biomass accumulation and culture viability during prolonged retentostat cultivation. **a** Predicted biomass accumulation profile (*line*), measured biomass dry weight concentrations (*open symbols*), and viable biomass concentration (*closed symbols*) from four replicate retentostat cultures. **b** Culture viability estimated by flow cytometric analysis of propidium iodide-stained cells (*closed* *symbols*), and viability estimated from colony-forming unit counts (*open* *symbols*)

In four independent retentostat cultures, biomass accumulated reproducibly over a period of ca. 20 days. The final biomass concentrations were ca. threefold higher than those in the preceding chemostat culture (Fig. 3a). However, the experimentally observed biomass accumulation was substantially higher than predicted from model simulations (Fig. 3a). One factor that might contribute to this apparent discrepancy was the biomass viability which, in the model simulations, was assumed to remain at 100 % throughout the retentostats experiments. Indeed, flow-cytometric analysis of cellular integrity indicated that, over 20 days of retentostat cultivation, culture viability decreased to ca. 85 % (Fig. 3b). Colony-forming unit counts confirmed that ca. 70 % of the cells in the population were able to sustain growth after 20 days in retentostat culture. This apparent loss of cells’ capacity to divide contrasted with the retention of cellular integrity and has been previously reported for anaerobic retentostat cultures [15]. It may result from various factors, such as the irreversible degradation of macromolecules necessary for duplication, but may also result from loss of reproductive capacity during CFU plating assays. To prevent the risk of underestimating culture viability, viable biomass concentrations were therefore calculated based on flow cytometry-based viability assays (Fig. 3a). Based on these observations, a low but significant death rate of 4.7·10^−4^ h^−1^ was calculated. However, correcting for viability only explained part of the difference between the observed and modelled biomass accumulation profiles.

**Fig. 4:**
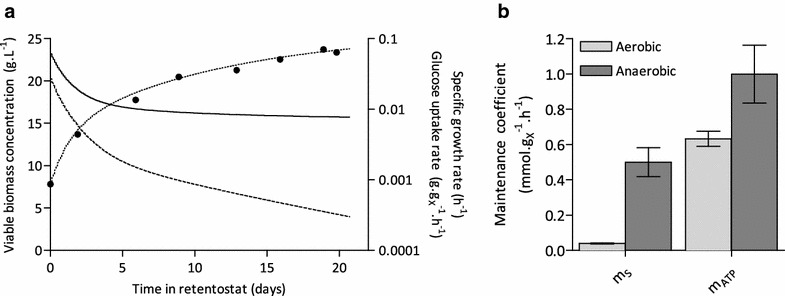
Growth kinetics and m_S_ in retentostat cultures. **a** Specific glucose-consumption rate (q_S_, *solid line*) and specific growth rate (µ, *dashed line*) calculated by non-linear regression of the accumulation of viable- and total biomass over time (see “Methods” section). The *closed symbols* and *dotted line* represent the viable biomass and linear regression of the viable biomass, respectively. Data are shown for a single representative retentostat experiment. **b** Glucose and ATP requirements for maintenance (m_S_ and m_ATP_, respectively) of aerobic and anaerobic retentostat cultures (anaerobic data obtained from [12]). The aerobic m_ATP_ was calculated based on a P/O ratio of respiring *S. cerevisiae* cultures of 1.0 [23]

As mentioned above, the exact value of m_S_ is expected to have a strong impact on biomass accumulation profiles in retentostat cultures. Assuming specific growth-rate independent maintenance, the aerobic m_S_ was estimated from the calculated specific growth rate and glucose consumption rates of *S. cerevisiae* in the aerobic retentostats, using biomass concentrations corrected for viability (Fig. 4a). During 20 days of retentostat cultivation, specific growth rates in all four replicate experiments decreased from 0.025 h^−1^ in steady-state to values below 8·10^−4^ h^−1^, representing doubling times of over 36 days. The average specific glucose-consumption rate converged to 0.039 ± 0.003 mmol g_X_^−1^ h^−1^, representing the cellular substrate requirement exclusively necessary for maintenance energy purposes (Fig. 4b). Considering an in vivo P/O ratio in *S. cerevisiae* of 1.0 [23], the aerobic ATP requirement of *S. cerevisiae* for maintenance (m_ATP_) calculated from these experiments was 0.63 ± 0.04 mmol ATP g_X_^−1^ h^−1^. This value is ca. 30 % lower than the m_ATP_ previously estimated from anaerobic retentostats cultures [12] (Fig. 4b).

### Transcriptional reprogramming in aerobic retentostats: involvement of ‘growth-rate responsive’ genes

**Fig. 5:**
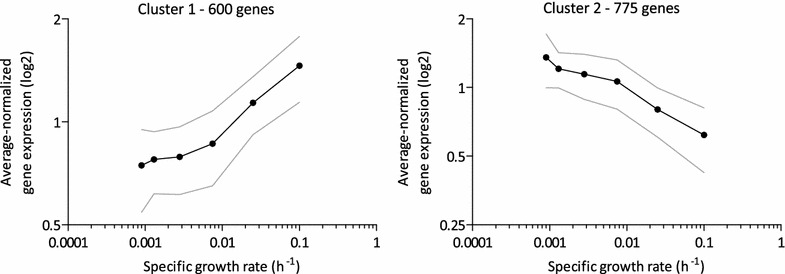
K-mean clustering of the 1375 genes with significant growth-rate dependent expression profiles. Retentostat data were combined with data from aerobic glucose-limited chemostats grown at µ = 0.10 h^−1^ (see “Methods” section). The p value threshold for significant differential expression was set to 0.01. For each cluster, averaged-normalized expression values are depicted as a function of specific growth rate (see “Methods” section). *Grey dotted lines* show the standard deviation of averaged expression values

Over the course of the aerobic retentostat experiments, 1375 genes (ca. one-fifth of the genome) were differentially expressed. In comparison, aerobic batch cultures transitioning from exponential growth, through a post-diauxic phase, into stationary phase, resulted in 1690 differentially expressed genes (using the same analysis software and statistical criteria as in the present work, Additional file 1) [34]. One third (458 genes) of the 1375 genes identified in the present retentostat dataset overlapped with the aerobic batch dataset. The 1375 differentially expressed genes identified in the present study could be separated in two clusters with clear, specific-growth-rate dependent transcript profiles (Fig. 5). Cluster 1 harboured 600 genes whose expression displayed a positive correlation with specific growth rate (Fig. 5). As anticipated, this cluster showed an overrepresentation of genes involved in typical growth-related processes, including protein, ribosome, amino acid, nucleotide and lipid biosynthesis (Table 1). Consistent with this observation, cluster 1 also showed an overrepresentation of genes whose expression is controlled by transcription factors that are involved in this response: Fhl1, Rap1 and Sfp1, Gcn4 and Met32 (Table 1). Genes involved in sterol metabolism (including 15 of the 19 *ERG* genes involved in ergosterol synthesis) and pentose-phosphate pathway were also overrepresented in cluster 1. Cluster 2 grouped the remaining 775 differentially expressed genes, whose transcript levels showed a negative correlation with specific growth rate (Fig. 5). This cluster was most strongly enriched for genes involved in stress response, and more specifically targets of Skn7 and Cad1, as well as for genes involved in signal transduction and protein turnover (Table 1). Accordingly, cluster 2 was strongly enriched for targets of the stress-responsive transcription factor pair Msn2/Msn4 (55 out of 166 genes, p value 4·29 10^−11^) [30].

**Table 1:** Overrepresentation of functional categories among the two clusters of differentially expressed genes (see Fig. 5)

		Functional category	K^a^	N^b^	p value^c^
Cluster 1	MIPS^d^	*Protein synthesis*	138	511	4.98·10^−31^
		Ribosomal proteins	95	277	4.29·10^−29^
		Ribosome biogenesis	106	343	2.46·10^−28^
		Amino acid metabolism	69	243	3.31·10^−15^
		*Metabolism*	221	1530	5.87·10^−11^
		Metabolism of the aspartate family	26	64	1.75·10^−8^
		Metabolism of methionine	18	36	3.52·10^−7^
		Tetracyclic and pentacyclic triterpenes metabolism	16	36	2.84·10^−5^
		Purine nucleotide/nucleoside/nucleobase metabolism	22	66	4.58·10^−5^
		Nucleotide/nucleoside/nucleobase metabolism	48	230	4.81·10^−5^
		Isoprenoid metabolism	16	41	2.55·10^−4^
		Sulfur metabolism	7	8	3.51·10^−4^
		Sulfate assimilation	7	8	3.51·10^−4^
		Metabolism of the cysteine-aromatic group	23	80	4.79·10^−4^
		Aminoacyl-tRNA-synthetases	15	39	7.46·10^−4^
		*Energy*	58	360	1.69·10^−2^
		Pentose-phosphate pathway	10	24	2.20·10^−2^
	GO^d^	Translation	117	345	6.31·10^−36^
		Cellular amino acid biosynthetic process	44	101	1.10·10^−16^
		Ribosome biogenesis	46	178	1.13·10^−7^
		Oxidation reduction	60	270	1.24·10^−7^
		Metabolic process	76	389	2.49·10^−7^
		Steroid biosynthetic process	15	24	2.73·10^−7^
		Sterol biosynthetic process	15	28	5.46·10^−6^
		Methionine biosynthetic process	16	32	6.13·10^−6^
		Maturation of SSU-rRNA	22	62	2.28·10^−5^
		Sulfate assimilation	9	11	3.49·10^−5^
		rRNA processing	43	195	8.44·10^−5^
		Methionine metabolic process	10	15	1.36·10^−4^
		Lipid biosynthetic process	18	52	7.16·10^−4^
		Ergosterol biosynthetic process	7	9	2.66·10^−3^
	TF^d^	*FHL1*	75	208	1.64·10^−24^
		*RAP1*	51	145	1.31·10^−15^
		*SFP1*	20	50	1.41·10^−6^
		*GCN4*	37	182	8.70·10^−4^
		*HAP1*	27	120	2.60·10^−3^
		*MET32*	9	24	4.06·10^−2^
Cluster 2	MIPS^d^	*Unclassified proteins*	194	1140	4.13·10^−5^
		Oxidative stress response	21	56	7.17·10^−4^
		*Cell rescue, defense and virulence*	101	558	9.06·10^−3^
		Degradation of polyamines	5	5	1.98·10^−2^
		*Energy*	70	360	2.14·10^−2^
		Cellular communication	50	239	4.60·10^−2^
		Cellular signalling	44	202	4.71·10^−2^
	GO^d^	Signal transduction	24	74	4.64·10^−3^
		Protein amino acid phosphorylation	36	141	1.11·10^−2^
		Proteasomal ubiquitin-dependent protein catabolic process	9	16	3.95·10^−2^
		Oxidation reduction	56	270	3.96·10^−2^
		Negative regulation of gluconeogenesis	7	10	4.55·10^−2^
	TF^d^	*MSN2/MSN4* ^e^	55	166	4.29·10^−11^
		*SKN7*	43	175	6.59·10^−4^
		*YAP7*	36	152	9.78·10^−3^
		*CAD1*	12	32	4.40·10^−2^

^a^Number of genes present in both the cluster and the functional category

^b^Total number of genes in the functional category

^c^A Bonferroni corrected p value cut-off of 0.05 was used and p values indicate the probability of finding the same number of genes in a random set

^d^Functional categories originate from the Munich Information Centre for Protein Sequences (MIPS), Gene Ontology (GO) or transcription factor binding datasets (TF) described in the “Methods” section

^e^MSN2/4 transcription factor dataset originates from [30]

A positive correlation with specific growth rate of the expression levels of genes involved in anabolic processes and a negative correlation of those of stress-responsive genes, was previously shown in aerobic chemostat cultures grown at specific growth rates of 0.05 h^−1^ and above [31–33]. Clusters 1 and 2 showed a substantial overlap with these previously identified sets of growth-rate responsive genes (Additional file 2).

To investigate how cellular responses to near-zero growth rates differed between aerobic and anaerobic cultures, we compared transcriptome data from the present study with those obtained in a previous transcriptome analysis of anaerobic retentostats of the same *S. cerevisiae* strain [14]. Anaerobic retentostat cultivation yielded 2661 differentially expressed genes, based on the same range of specific growth rates and applying the same statistical criteria as in the present study. This number of genes is almost twofold higher than observed in the aerobic retentostats (Additional file 3). Synthetic medium, pH and temperature in the anaerobic retentostats were the same as those used in the present study, except for the addition of the anaerobic growth factors Tween-80 (a source of oleic acid) and ergosterol in the previous study.

**Fig. 6:**
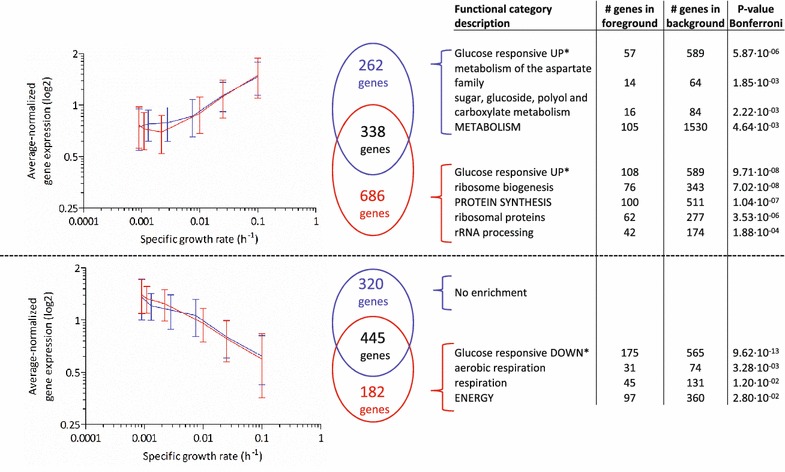
Comparison between aerobic and anaerobic growth-rate dependent gene expression at near-zero growth rates. Transcriptome datasets of aerobic (*blue*) and anaerobic (*red*) experiments covered a specific growth rate range between 0.1 h^−1^ and values below 0.001 h^−1^, with an equal number of data points ([14] and Fig. 5). The p value threshold for significant differential expression was 0.01. Overlapping and exclusive gene groups within the clusters, presented as Venn diagrams, were mined for overrepresentation of genes involved in specific functional categories with a Bonferroni-corrected p value threshold of 0.05 (see “Methods” section). Genes in the foreground represent number of genes present in both the cluster and the functional category, Genes in the background represent the total number of genes in the functional category. *Asterisk* Glucose-responsive gene sets are derived from [65]

Differences in the responses of anaerobic and aerobic retentostats were investigated by identifying genes that showed a specific transcriptional response to near-zero growth rates in either aerobic or anaerobic retentostats (Fig. 6). Among 182 genes whose expression increased at extremely low growth rates in anaerobic retentostat cultures, only functional categories related to aerobic respiration, were significantly enriched (Fig. 6). 31 out of 74 genes involved in the cellular function aerobic respiration were specifically up-regulated in anaerobic retentostats, including 8 *COX* genes, which encode subunits of the mitochondrial inner-membrane cytochrome c oxidase. Genes involved in this functional category were not overrepresented among the responsive genes identified in aerobic retentostat cultures, indicating that up-regulation of respiration-related genes is a specific adaptation to anaerobic slow growing and/or aging cultures. Among the 686 genes whose expression showed a reduced expression at near-zero growth rates under anaerobic conditions, functional categories related to protein synthesis were significantly enriched (Fig. 6).

### Extreme heat-shock tolerance of yeast cells grown in aerobic retentostats

**Fig. 7:**
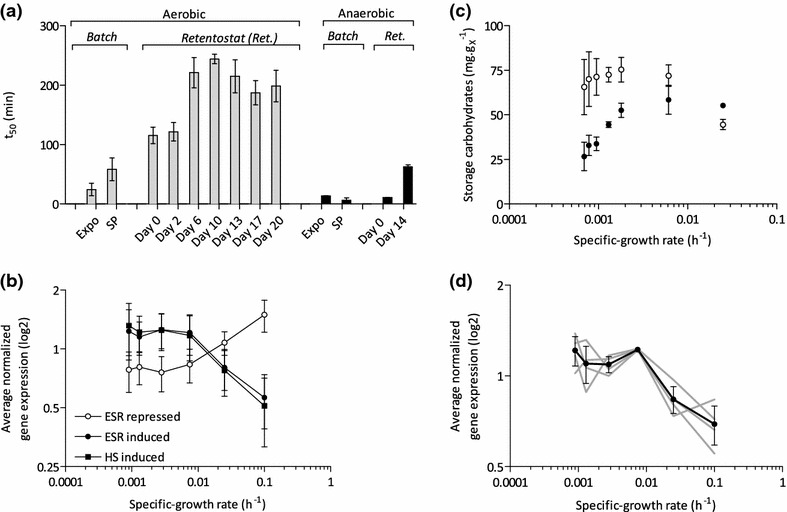
Heat-shock tolerance of aerobic and anaerobic retentostat and batch cultures. **a** Data on heat shock tolerance of anaerobic retentostat cultures and from batch cultures are taken from previous studies [13, 34]. Batch cultures were characterized during the exponential growth phase (expo) and after ca. 2 h in stationary phase (SP) [34]. t_50_ represents incubation time at 53 °C at which 50 % of the initial viable cell population was still alive. **b** Transcript levels of genes that exhibit a significant growth-rate dependent expression in retentostat, and are also known to respond to environmental stress and heat shock according to [30, 35]. **c** Cellular contents of trehalose (*open symbols*) and glycogen (*closed symbols*) during prolonged retentostat cultivation. **d** Average-normalized expression profiles of genes involved in trehalose metabolism (see “Methods” section)

Studies in aerobic chemostats, anaerobic retentostats and aerobic stationary-phase batch cultures showed that slow growth of *S. cerevisiae* increases its stress tolerance, most often measured as its ability to survive exposure to high temperatures [13, 22, 34]. The aerobic chemostat cultures, grown at a specific growth rate of 0.025 h^−1^, which preceded the retentostat cultures were already remarkably heat-shock tolerant, with 50 % of the population surviving a 115 min exposure to a temperature of 53 °C (Fig. 7a). After 10 days of retentostat cultivation, when the specific growth rate had decreased below 0.001 h^−1^, this t_50_ had increased to 4 h. This t_50_ value is approximately fourfold higher than previously described for extremely heat-shock tolerant cultures, such as aerobic stationary-phase and anaerobic retentostat cultures (Fig. 7a). To the best of our knowledge, this heat-shock tolerance is the highest measured to date in *S. cerevisiae*.

In previous studies, a high heat-shock tolerance of *S. cerevisiae* was found to correlate with increased transcript levels of many known stress-responsive genes [13, 34]. Consistent with these earlier observations, transcript levels of *Msn2/4* gene targets, as well as genes that were previously shown to be responsive to environmental stresses (ESR induced: 110 out of 281, p value 1.17·10^−30^; ESR repressed: 170 out of 563, p value 3.31·10^−48^) or to heat shock in an Msn2/4-independent manner (125 out of 427, p value 3.11·10^−21^), correlated with specific growth rate and, therefore, with heat-shock tolerance in the aerobic retentostat cultures (Fig. 7b) [30, 35]. Heat-shock proteins function as chaperones that prevent aggregation of thermally damaged proteins, unfold them, or mark them for degradation [29]. Of 76 genes known to encode heat-shock proteins, seven showed increased mRNA levels at near-zero growth rates (*SSA3*, *HSP26*, *HSP42*, *XDJ1*, *CWC23*, *EUG1* and *HSP60*) [29]. Disaggregation and (re)folding activities of heat-shock proteins are ATP dependent and maintaining intracellular ATP levels has been shown to be crucial for heat-shock survival of stationary-phase batch cultures [34, 36]. High contents of the intracellular carbohydrate storage materials trehalose and glycogen (>10 % of biomass dry-weight, Fig. 7c) may have contributed to the extreme heat-shock tolerance of yeast cells grown in aerobic retentostat cultures by supplying the ATP that is required to combat heat stress (Fig. 7c). In addition to intracellular trehalose concentrations, expression of the trehalose-metabolism related genes *TPS1*, *TPS2*, *ATH1* and *NTH1* increased substantially when retentostat cultures approached near-zero growth rates (Fig. 7c, d) [36, 37]. The strong increase of intracellular trehalose concentrations in the aerobic retentostat cultures represents a marked difference with published data on anaerobic retentostats, in which intracellular trehalose levels were low and glycogen was the predominant storage carbohydrate [14].

### Aerobic retentostat cultures retain a high glycolytic capacity at near-zero growth rates

**Fig. 8:**
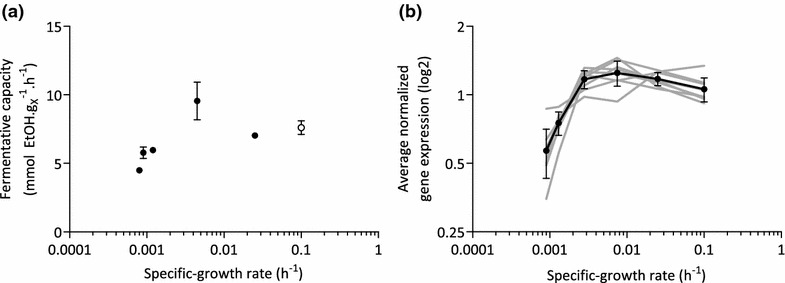
Fermentative capacity and expression levels of glycolytic genes in *S. cerevisiae* at near-zero growth rates. **a** Fermentative capacity, measured off-line as the specific rate of ethanol formation upon exposure of anaerobic cell suspensions to excess glucose. Fermentative capacity assays were performed on independent duplicate retentostat cultures, sampled at different time points. The *open symbol* corresponds to data from [28]. **b** Log2 average-normalized gene expression of *HXK2, FBA1, PGK1, GPM1, ENO1, ENO2, PYK1*, and *PDC1* during retentostat cultivation, plotted as a function of specific growth rate (see “Methods” section)

Glycolysis, together with glucose transport, pyruvate decarboxylase and alcohol dehydrogenase, represents the pathway for alcoholic fermentation in *S. cerevisiae*. Respiratory cultures of this yeast maintain a high capacity for fermentative metabolism (fermentative capacity), which allows *S. cerevisiae* to rapidly increase its glycolytic flux in response to, for example, oxygen depletion and/or exposure to high sugar concentrations [38–40]. In aerobic glucose-limited chemostat cultures of the *S. cerevisiae* CEN.PK113-7D strain, fermentative capacity is essentially growth-rate independent at specific growth rates between 0.05 and 0.3 h^−1^, [28]. The fermentative capacity of 7.5 mmol ethanol g_X_^−1^ h^−1^ measured in the aerobic chemostats (D = 0.025 h^−1^, Fig. 8a), matched well with the fermentative capacity found previously at these higher specific growth rates [28]. After 18 days of aerobic retentostat cultivation, a significantly lower (p value < 0.05) fermentative capacity of 4.5 mmol ethanol g_X_^−1^ h^−1^ was measured (Fig. 8a). The corresponding glucose-consumption rate was 45-fold higher than the specific rate of glucose consumption measured in the retentostat at this time point.

The decrease of the fermentative capacity in the aerobic retentostats coincided with a decrease of the transcript levels of a subset of glycolytic genes, some of which encoded major isoforms of glycolytic enzymes [41]. Expression levels of *HXK2* encoding hexokinase 2, first step in glycolysis*, FBA1* encoding the single fructose-bisphosphate aldolase*, PGK1* encoding the single phosphoglycerate kinase*, GPM1* encoding the major phosphoglycerate mutase*, ENO1* and *ENO2* paralogs encoding the two yeast enolases*, PYK1* also known as *CDC19*, encoding the major pyruvate kinase, last step in glycolysis, and *PDC1* encoding pyruvate decarboxylase 1 responsible for the first step in the fermentative pathway leading to ethanol, were stable during the initial phase of the retentostat cultures, but significantly and substantially decreased at growth rates below 0.002 h^−1^ (Fig. 8b). Pair-wise comparison of transcriptome data for day 0 and day 16 of the retentostats (corresponding to specific growth rates of 0.025 and 0.0009 h^−1^, respectively) showed at least a twofold difference in expression levels of *HXK2*, *PGK1*, *GPM1*, *ENO2* and *PDC1*. Overrepresentation of binding sites for Rap1/Gcr1 in their promoter regions suggests that these transcription factors may be involved in their transcriptional regulation at near-zero growth rates. This hypothesis is further supported by the observation that 51 of the 145 gene targets of the transcription factor Rap1 were part of cluster 1 (Fig. 4, Table 1). While we cannot exclude that decreased glucose transport was also involved in the reduction of fermentative capacity, no difference was observed in *HXT* gene expression at near-zero growth rates.

## Discussion

### Estimation of maintenance-energy requirements from aerobic retentostats

Initially developed by microbial ecologists to explore the ‘twilight zone’ between exponential growth and starvation [17, 19–21, 42, 43], the retentostat has recently seen a revival in studies on industrial microorganisms [18]. A key advantage of retentostat cultivation for application-inspired research is that it enables an accurate, quantitative estimation of microbial maintenance-energy requirements [18]. The conventional method for determining m_S_ does not measure, but estimates the specific rate of energy-substrate consumption in non-growing cultures, based on extrapolation of measurements on chemostat cultures that are actively growing (often at specific growth rates of 0.05 h^−1^ and above). Since, at these specific growth rates, substrate consumption for maintenance is relatively small as compared to the overall consumption rate of the energy substrate, m_S_ values calculated via this procedure are sensitive to small measurement errors [3, 18]. Moreover, chemostat-based estimation of m_S_ is based on the assumption that this parameter is growth-rate independent. Studies on several prokaryotes have shown that this assumption is not always valid and that, at extremely slow growth rates, several bacteria downregulate ATP turnover and thereby reduce substrate consumption for maintenance [20, 44].

Even at extremely low specific growth rates, the energetics of aerobic, glucose-limited retentostat cultures of *S. cerevisiae* could be adequately described with a growth-rate independent m_S_. The same conclusion was drawn earlier for anaerobic, glucose-limited retentostat cultures of this yeast [12]. The value of m_S_ estimated from the aerobic retentostat cultures was 0.039 mmol glucose g_X_^−1^ h^−1^. There are surprisingly few, invariably chemostat-based, estimates of the m_S_ of aerobically grown *S. cerevisiae*. Four decades ago, Rogers and Stewart [45] calculated an m_S_ of 0.07 mmol glucose g_X_^−1^ h^−1^ based on aerobic chemostat cultures of a diploid *S. cerevisiae* strain, grown at pH 5.5 on a complex medium. This value is 75 % higher than the m_S_ found in the present study. Recently, based on aerobic chemostat cultures of the same haploid *S. cerevisiae* strain that is used in the present study, grown at pH 5.5, we estimated an m_S_ that was even 2.5-fold higher than calculated from the aerobic retentostats [3]. It should, however, be noted that the latter study used a growth medium that contained high concentrations of copper, which may have negatively affected cellular energetics.

Based on an assumed P/O ratio of 1.0 [23, 29, 46, 47], the maintenance requirement for ATP (m_ATP_) estimated from the aerobic retentostat cultures was 0.63 mmol ATP g_X_^−1^ h^−1^, a value 35 % lower than previously estimated based on anaerobic retentostat cultures of the same *S. cerevisiae* strain [12]. One possible explanation for this difference is that anaerobic growth indeed results in a higher m_S_, for instance as a result of increasing proton leakage across membranes due to the presence of the fermentation products ethanol and acetic acid. Additionally, the anaerobic growth factor oleic acid, which is added to anaerobic chemostat media as the oleate ester Tween-80, has been shown to negatively affect growth energetics [25]. Alternatively, the assumed P/O value of 1.0 might be wrong. However, if this were the sole reason for the observed difference, the actual P/O ratio would have to be close to 1.7, which falls outside the range of estimates for this parameter from several quantitative physiological studies on *S. cerevisiae* [23, 29, 46, 47]. The lower m_ATP_ under aerobic conditions, makes it unlikely that the presence of oxygen or generation of ROS in respiration increases maintenance-energy requirements.

Maintenance-energy requirements are well known to depend on growth conditions, for example on the presence of weak organic acids [48–50], and may additionally be strain dependent. The present study demonstrates that retentostat cultivation offers a robust way to estimate m_S_. The large impact of this parameter on the performance of large-scale industrial fed-batch processes provides a strong impetus for using this, somewhat technically demanding, approach for determining and comparing maintenance-energy requirements of different production hosts under carefully controlled, industrially relevant experimental conditions.

### Extreme heat-shock tolerance of aerobic retentostat cultures

In industrial processes, yeast cells face a variety of stresses, including high concentrations of CO_2_ and other products, inhibitors in low-grade media, fluctuations in nutrient availability (e.g. during biomass recycling and ‘repitching’ in beer fermentation) and high as well as low temperatures [51, 52]. Here we show that aerobic retentostat cultures of *S. cerevisiae* grown at near-zero growth rates acquire an extreme resilience to heat shock. We recently reported that stationary-phase, glucose-grown aerobic batch cultures of *S. cerevisiae* are much more heat-shock tolerant than the corresponding anaerobic cultures [34]. This difference was tentatively attributed to the much faster transition from exponential growth to nutrient depletion in anaerobic batch cultures, which do not exhibit the second, slow growth phase on ethanol that is characteristic for aerobic glucose-grown batch cultures of *S. cerevisiae*. The hypothesis that this fast transition prevented a full induction of heat-shock tolerance was consistent with the earlier observation that anaerobic retentostat cultures, which undergo a slow transition to near-zero growth rates, exhibit a much higher heat-shock tolerance than anaerobic stationary-phase batch cultures [34]. The present study shows that, despite a very similar ‘conditioning’, the heat-shock tolerance of aerobic retentostat cultures is much more pronounced than in anaerobic retentostats (four to fivefold higher t_50_, Fig. 7a). Indeed, to our knowledge, the heat-shock tolerance of the aerobic retentostat cultures is the highest reported to date for *S. cerevisiae*. These observations indicated that a smooth transition from exponential growth to (near-)zero growth in aerobic cultures provides an optimal conditioning for heat-shock tolerance in this yeast. Further research is required to assess whether this conclusion can be extended to include conditioning for other industrially relevant stresses, such as freezing/drying, osmotic stress and oxidative stress.

Intracellular concentrations of trehalose and regulation of genes involved in its metabolism showed a remarkable correlation with the different levels of heat-shock tolerance in aerobic retentostats. Trehalose can act as an energy reserve, and has also been proposed to be directly involved in heat shock resistance [53–55]. However, recent evidence suggests that secondary, so called ‘moonlighting’ functions of the trehalose-6-phosphate synthase Tps1, rather than trehalose itself, contribute to cell integrity during heat shock [36]. Additionally, different expression levels of other stress-induced proteins and different membrane composition, resulting from the inability of anaerobic cultures to synthetize unsaturated fatty acid and sterols, may contribute to the different heat-shock tolerance of aerobic and anaerobic *S. cerevisiae* cultures [25, 56, 57].

### *S. cerevisiae* down-regulates glycolytic gene expression but maintains a high fermentative capacity at near-zero growth

Protein synthesis is the single most ATP-intensive process in living cells [58], and especially proteins with relatively high expression levels and short turnover times are expected to represent a significant metabolic burden to cells grown under severely calorie-restricted retentostat cultivation regimes. In actively growing *S. cerevisiae* cultures, glycolytic enzymes make up a significant fraction of the total cellular protein [59]. The half-life of most glycolytic proteins in *S. cerevisiae* grown in glucose-excess conditions range between 5 and 20 h, excluding Tdh1, Tdh2, Gpm2, and Eno1, for which half-lives of over 100 h have been determined [60]. These reported half-lives are much lower than the amount of time that the cells reside in retentostat; protein turnover of glycolytic proteins could therefore significantly contribute to the energy requirements of *S. cerevisiae* at near-zero growth. Under many conditions, this yeast exhibits a large overcapacity of the glycolytic pathway. Indeed, a substantial loss of fermentative capacity has previously been observed during prolonged cultivation of *S. cerevisiae* in aerobic, glucose-limited chemostat cultures (50 % after 100 generations) [61]. This loss was attributed to mutations that reduced the metabolic burden of synthesizing large amounts of glycolytic proteins. Although retentostat-grown cells retained a high glycolytic capacity, this decreased by ca. 40 % at extremely low specific growth rates. It is, however, unlikely that evolutionary adaptation caused this reduction in glycolytic capacity, since the average number of generations in the retentostat experiments was approximately three as a consequence of the biomass retention. Instead, the reduced mRNA levels of several glycolytic genes suggest a transcriptional downregulation of this key pathway at extremely low growth rates. Furthermore, glycolytic genes *PGK1* and *PYK1* that are considered to be constitutively expressed at high levels [62], displayed ca. twofold reduced transcript levels at near-zero growth (Fig. 8), and shows that glycolytic promoters for the expression of (heterologous) proteins should be carefully selected.

### Impact of oxygen availability on transcriptional reprogramming at near-zero growth rates

The specific growth rate profiles and experimental conditions employed in the aerobic retentostat cultures very strongly resembled those applied in a previous study on anaerobic retentostats of the same *S. cerevisiae* strain. Gene sets that showed a transcriptional response in these retentostat experiments showed a strong overrepresentation of growth-rate responsive genes identified by Fazio et al. [63]. These authors used chemostats, grown at specific growth rates of 0.03 h^−1^ and higher, to explore transcriptional responses under different aerobic and anaerobic nutrient-limitation. Of the set of 268 growth-rate-responsive genes identified in their study, 115 genes were also found to show growth-rate dependent expression at the very low specific growth rates studied in the aerobic and anaerobic retentostats (Additional file 3). Despite this clear overlap in transcriptional responses, the number of genes that showed a transcriptional response to the shift to near-zero growth rates was twofold higher in the anaerobic retentostats than in the aerobic retentostats. As discussed above, ATP yields from respiratory and fermentative glucose dissimilation differ by a factor of approximately 8. As a consequence, at any specific growth rate, specific rates of glucose consumption (q_S_) in anaerobic glucose-limited cultures are higher than in the corresponding aerobic cultures. For example, at a specific growth rate of 0.025 h^−1^, the q_S_ in anaerobic glucose-limited chemostat cultures (2.3 mmol g_X_^−1^ h^−1^ (Additional file 4, [12]), was ca. eightfold higher than in the corresponding aerobic chemostat cultures [0.3 mmol g_X_^−1^ h^−1^ (Additional file 4)]. Simple monod kinetics [64] predict that this difference should also be reflected in the concentration of the growth-limiting nutrient. Indeed, residual glucose concentrations in these anaerobic and aerobic cultures were 0.3 and 0.07 mM, respectively (Additional file 4 and [14]). The consequence of these differences is that aerobic and anaerobic retentostat cultures operate in a different range of residual glucose concentrations. Concomitantly, a set of previously identified glucose-responsive transcripts were specifically overrepresented under anaerobiosis among genes which were transcriptionally up and down regulated with specific growth rate in retentostat cultures (Fig. 6) [65]. This comparison identifies differences in glucose concentration as a major cause of the different transcriptome profiles of aerobic and anaerobic retentostat cultures.

## Conclusion

Glucose-feeding regimes of retentostat cultures were optimized by model simulations to enable a first characterization of glucose-limited, aerobic cultures of *S. cerevisiae* during a smooth transition to extremely low specific growth rates. Quantitative analysis of these retentostats enabled the most accurate estimation to date of the growth-rate-independent maintenance-energy requirement of this yeast. Aerobic, glucose-limited retentostat cultures of *S. cerevisiae* showed a high viability, an extremely high heat-shock tolerance and retained an overcapacity of the fermentative pathway, thus illustrating the potential of this yeast to be developed for robust product formation in the absence of growth. This study shows that retentostat cultures, although technically demanding, offer unique possibilities for quantitative analysis of industrially relevant aspects of microbial physiology.

## Methods

### Yeast strain and shake-flask cultivation

The prototrophic strain *S. cerevisiae* CEN.PK113-7D (*MATa, MAL2*-*8c, SUC2*; [66, 67]) was used in this study. Stock cultures were grown in 500 mL shake flasks containing 100 mL YPD medium (10 g L^−1^ Bacto yeast extract, 20 g L^−1^ Bacto peptone and 20 g L^−1^d-glucose). After addition of glycerol (20 % v/v) to early stationary-phase cultures, 2 mL aliquots were stored at −80 °C. Shake-flask precultures for chemostat experiments were grown in 500 mL shake flasks containing 100 mL of synthetic medium, set to pH 6.0 with 2 M KOH prior to autoclaving and supplemented with 20 g L^−1^d-glucose [49]. These shake-flask cultures were inoculated with 2 mL of frozen stock culture and incubated in an orbital shaker at 200 rpm and at 30 °C.

### Chemostat cultivation

Chemostat cultivation was performed in 2-liter bioreactors (Applikon, Delft, the Netherlands) equipped with a level sensor to maintain a constant working volume of 1.4 L. The culture temperature was controlled at 30 °C and the dilution rate was set at 0.025 h^−1^ by controlling the medium inflow rate. Cultures were grown on synthetic medium, prepared as described previously [49] but with the following modifications: the glucose concentration was increased to 20 g L^−1^ glucose (C_S,MC_), the amount of trace-element and vitamin solutions were increased to 1.5 and 2 mL L^−1^ respectively [49], and 0.25 g L^−1^ Pluronic 6100 PE antifoaming agent (BASF, Ludwigshafen, Germany) was used. Fresh medium was supplied to the bioreactor from a 3-liter stirred mixing vessel (Applikon, Delft, The Netherlands) whose working volume (*V*_*S*_) of 1.2 L was controlled by a level sensor and which was stirred continuously at 500 rpm. The mixing vessel was equipped with a sampling port. Medium was added to the mixing reactor by automatic addition from a medium reservoir, with a flow rate (ɸ_V_) of 35 mL h^−1^ corresponding to the dilution rate in the bioreactor. Cultures were sparged with air (0.5 vvm) and stirred at 800 rpm. Culture pH was kept constant at 5.0 by automatic addition of 10 % NH_4_OH. Chemostat cultures were assumed to be in steady state when, after at least 6 volume changes, culture dry weight and the specific carbon dioxide production rates changed by less than 3 % over 2 consecutive volume changes. Steady-state carbon recoveries of chemostat cultures included in this study were above 98 %. Chemostat experiments performed at a dilution rate of 0.10 h^−1^ were performed as described above, with the following modifications: cultures were grown on synthetic medium [49] without modifications, with 7.5 g L^−1^ glucose, 1 mL L^−1^ trace elements solution, and 1 mL L^−1^ vitamin stock solution.

### Retentostat

After reaching a steady-state in chemostat cultures, the retentostat phase was started by switching the reactor effluent to an outflow port equipped with an autoclavable Applisense filter assembly (Applikon), consisting of a hydrophobic polypropylene filter with a pore size of 0.22 µm and a stainless steel hollow filter support. Prior to autoclaving, the filter was wetted by overnight incubation in 96 % ethanol, and subsequently rinsed with a phosphate buffer saline solution (containing per 1 L demi-water: 8 g NaCl, 0.2 g KCl, 1.44 g Na_2_HPO_4_, 0.24 g KH_2_PO_4_, and HCl to adjust the final pH to 7.4). To control biomass accumulation, the medium reservoir connected to the mixing vessel (see above) was exchanged for a reservoir containing standard synthetic medium [49] supplemented with 7.5 g L^−1^ glucose (C_S,MR_) and 0.25 g L^−1^ pluronic 6100 PE antifoam. Consequently, the concentration of growth-limiting substrate glucose entering the bioreactor [C_S,in_ in (g L^−1^)] decreased over time [*t* in (h)] according to Eq. 3.

3 $$C_{S,in} = \left( {C_{S,MC} - C_{S,MR} } \right) \cdot e^{{\frac{{ - \phi_{V} t}}{{V_{S} }}}} + C_{S,MR}$$

In this equation, C_S,MC_ and C_S,MR_ correspond to the glucose concentrations in the medium entering the mixing vessel during the chemostat and the retentostat phase respectively. During retentostat cultivaton, culture pH was controlled by automatic addition of 2 M KOH. Sampling frequency and sample volume were minimized to limit the impact of sampling on biomass accumulation inside the reactor. Culture purity was routinely checked by microscopy and by plating on synthetic medium agar containing 20 g L^−1^ glucose and 20 mM LiCl [68]. Full biomass retention was confirmed by plating filtered effluent on YPD containing 2 % (w/v) agar.

### Predicting retentostat growth kinetics

Operational conditions to enable a smooth transition of the retentostat cultures to near-zero growth rates, were defined with a mathematical model that simulates growth kinetics of yeast during aerobic retentostat cultivation (See Additional files 5, 6, 7). Essentially, the mass balance equation for biomass (Eq. 4) was solved using MATLAB^®^ ode45 solver, by incorporating the substrate mass balance (Eq. 5), with the Pirt relation [69] (Eq. 1; Fig. 1e).

4 $$\frac{{dC_{X} }}{dt} = \mu C_{X}$$

5 $$\frac{{dC_{S} }}{dt} = \frac{{\upphi_{V} }}{V}\left( {C_{S,in} - C_{S} } \right) - q_{S} C_{X}$$

In these equations, C_X_ (g L^−1^) is the biomass concentration in the retentostat, µ (h^−1^) is the specific growth rate, C_S_ (g L^−1^) is the residual substrate concentration, C_S,in_ (g L^−1^) is the substrate concentration in the feed, $\frac{{\upphi_{\text{V}} }}{\text{V}}$ (h^−1^) is the dilution rate, and q_S_ (g g_X_^−1^ h^−1^) is the biomass-specific glucose consumption rate. The specific substrate consumption rate can be described by the Pirt relation (Eq. 1), in which Y_X/S_^max^ [g g^−1^] is the maximum biomass yield on glucose, and m_S_ (g g_X_^−1^ h^−1^) is the maintenance coefficient. Because retentostats were glucose limited and C_S,in_ ≫ C_S_, the glucose concentration in the retentostat was assumed to be in a pseudo-steady state such that dC_S_/dt ≈ 0.

To run simulations, the model required inputs for variables V (bioreactor volume) (L), ɸ_V_ (flow rate) (L h^−1^), C_S,MC_ (g L^−1^), C_S,MR_ (g L^−1^), and V_S_ (L), and generated time-dependent profiles for biomass accumulation, glucose concentration in the feed, specific glucose consumption rates, and specific growth rates for a range of m_S_ values. The final operational conditions chosen for the retentostat experiments are indicated in Fig. 2.

### Regression analysis of biomass accumulation in retentostat

The maintenance-energy requirements and biomass-specific death rate of *S. cerevisiae* in aerobic retentostat were estimated from a least-squares regression analysis of data points for the biomass concentration (dry-weight) and the viable biomass concentration over time, using a MATLAB model (see Additional files 8, 9, 10, 11, 12). From these parameters, the specific growth rate and substrate consumption rates were derived. The curve shape was determined by the solution of the following ordinary differential equations with the smallest sum of square errors:

6 $$\frac{{dC_{X\_V} }}{dt} = \mu C_{X\_V} - k_{d} C_{X\_V}$$

7 $$\frac{{dC_{X\_d} }}{dt} = k_{d} C_{X\_V}$$

8 $$\frac{{dC_{S} }}{dt} = \frac{{\upphi_{V} }}{V}\left( {C_{S,in} - C_{S} } \right) - q_{S} C_{X\_V}$$

In these equations, C_X_V_ is the viable biomass concentration (g L^−1^), k_d_ is the death rate (h^−1^). Equation 1 was used to define the specific substrate consumption rate (q_S_).

The model required input for the biomass concentrations measured at different time points, and the following variables: V (L), ɸ_V_ (L h^−1^), C_S,MC_ (g L^−1^), C_S,MR_ (g L^−1^), V_S_ (L) and Y_X/S_^max^. A value for m_S_ was approximated using parameter estimation. The time-dependent change of q_S_ and µ during the course of the retentostat followed from the regression analysis (see Additional files 8, 9, 10, 11, 12). To respect small differences in operational variables per experiment, regression analyses were performed separately on each independent retentostat experiment.

### Determination of substrate, metabolites and biomass concentration

Prior to culture dry weight assays, retentostat samples were diluted in demineralized water. Culture dry weight was measured by filtering exactly 10 mL of an appropriate dilution of culture broth over pre-dried and pre-weighed membrane filters (pore size 0.45 µm, Gelman Science), which were then washed with demineralized water, dried in a microwave oven (20 min, 350 W) and reweighed. Supernatants were obtained by centrifugation of culture samples (3 min at 16,000×*g*) and analysed by high-performance liquid chromatography (HPLC) analysis on a Agilent 1100 HPLC (Agilent Technologies, Santa Clara, CA, USA), equipped with an Aminex HPX-87H ion-exchange column (BioRad, Veenendaal, The Netherlands), operated with 5 mM H_2_SO_4_ as the mobile phase at a flow rate of 0.6 mL min^−1^ and at 60 °C. Detection was by means of a dual-wavelength absorbance detector (Agilent G1314A) and a refractive-index detector (Agilent G1362A). Residual glucose concentrations in chemostat and retentostat cultures were analysed by HPLC after rapid quenching of culture samples with cold steel beads [70].

### Gas analysis

The exhaust gas from chemostat cultures was cooled with a condenser (2 °C) and dried with a PermaPure Dryer (model MD 110-8P-4; Inacom Instruments, Veenendaal, the Netherlands) prior to online analysis of carbon dioxide and oxygen with a Rosemount NGA 2000 Analyser (Baar, Switzerland).

### Viability

Small aliquots of culture broth (<1 mL) were sampled in a 10 mM Na-Hepes buffer (pH 7.2) with 2 % glucose. Cell numbers were determined with a Coulter counter using a 50 µm orifice (Multisizer II, Beckman, Fullerton, CA). Colony-forming units (CFU) in culture samples were quantified by triplicate plating of 10-fold dilution series in 0.1 % peptone on 2 % YPD agar plates. At least 150 colonies were counted after 2 days of incubation at 30 °C to calculate CFU. Viability was then calculated by comparing CFU counts with total cell counts. Additionally culture viability was assayed by propidium iodide (PI) staining (Invitrogen, Carlsbad, CA) by counting 10,000 cells on a Cell Lab Quanta SC MPL flow cytometer (Beckman Coulter, Woerden, Netherlands) as described previously [13]. PI intercalates with DNA in cells with a compromised cell membrane, causing a red fluorescence.

### Heat shock resistance assays

Samples from retentostat cultures were added to Isoton II diluent (Beckman Coulter, Woerden, Netherlands), pre-heated at 53 °C, to a final concentration of 10^7^ cells mL^−1^, and incubated at 53 °C for at least 200 min. Loss of viability was monitored by sampling at 20 min intervals. Samples were immediately cooled on ice and subsequently stained with PI and analysed by flow cytometry as described above. Heat-shock resistance was represented by t_50_, the incubation time at 53 °C that lead to a 50 % decrease in viability. To calculate t_50_, survival curves were fitted with a sigmoidal dose–response curve in Graphpad^®^ Prism, version 4.03.

### Glycogen and trehalose assays

1 mL broth was sampled from the retentostat or chemostat and immediately added to 5 mL of cold methanol (−40 °C), mixed and centrifuged (4400×*g*, −19 °C, 5 min). The supernatant was decanted and pellets were resuspended in 5 mL cold methanol, pelleted again and stored at −80 °C. Pellets were then resuspended and diluted in 0.25 M Na_2_CO_3_, and further processed as previously described [71]. Trehalose was directly measured by HPLC. Glucose released from glycogen was measured by HPLC after overnight incubation of samples at 57 °C with ɑ-amyloglucosidase (from *Aspergillus niger*, Sigma-Aldrich, Zwijndrecht, Netherlands).

### Fermentative capacity assays

Samples containing exactly 100 mg dry weight of biomass from retentostat cultures were harvested by centrifugation at 5000×*g* for 5 min, washed once, and resuspended in 10 mL fivefold concentrated synthetic medium (pH 6, [49]). Subsequently, these cell suspensions were introduced into a 100 mL reaction vessel maintained at 30 °C, which was kept anaerobic with a constant flow (10 mL min^−1^) of water-saturated CO_2_. After addition of 40 mL demineralized water and 10 min of pre-incubation, 10 mL glucose solution (100 g L^−1^) was added, and 1 mL samples were taken at 5 min intervals. After centrifugation, ethanol concentrations in supernatants were determined by HPLC. Fermentative capacity, calculated from the increase in ethanol concentration during the first 30 min of the experiments, was expressed as mmol ethanol produced (g of dry yeast biomass)^−1^ h^−1^. During the assay period, the increase in biomass concentration was negligible, and the increase in ethanol concentration was linear with time and proportional to the amount of biomass added.

### Transcriptome analysis

Microarray analysis was performed with samples from independent triplicate steady-state chemostat cultures and duplicate retentostat cultures of *S. cerevisiae* strain CEN.PK113-7D sampled at 5 time points, comprising a total dataset of 13 microarrays. Sampling for transcriptome analysis was carried out by using liquid nitrogen for rapid quenching of mRNA turnover [72]. Prior to RNA extraction, samples were stored in a mixture of phenol/chloroform and TEA buffer at −80 °C. Total RNA extraction, isolation of mRNA, cDNA synthesis, cRNA synthesis, labelling and array hybridization was performed as described previously [73], with the following modifications. The quality of total RNA, cDNA, aRNA and fragmented aRNA was checked using an Agilent Bioanalyzer 2100 (Agilent Technologies, Santa Clara, CA). Hybridization of labelled fragmented aRNA to the microarrays and staining, washing and scanning of the microarrays was performed according to Affymetrix instructions (EukGE_WS2v5).

The 6383 yeast open reading frames were extracted from the 9335 transcript features on the YG-S98 microarrays. To allow comparison, all expression data were normalized to a target value of 240 using the average signal from all gene features. To eliminate variation in genes that are essentially not expressed, genes with expression values below 12 were set to 12 and the genes for which the average expression was below 20 for all 13 arrays were discarded. The coefficient of variation of the mean transcript data of replicate retentostats was approximately 10 %, similar to the reproducibility usually observed in replicate steady state chemostat cultures [74]. The expression of housekeeping genes *ACT1, HHT2, SHR3, PDA1,TPI* and *TFC1* [75] remained stable for both strains at all tested growth rates (average coefficient of variation 11 ± 4 % see Additional file 13).

To perform a differential expression analysis based on gene expression profiles across the different growth rates, EDGE version 1.1.291 [76] was used with growth rate as covariate. Genes with expression profiles with a p value below 0.01 were considered to significantly correlate with growth rate, and were clustered with k-means clustering using consensus clustering (GenePattern 2.0, Broad Institute, [77]). For display of specific growth rate dependent expression profiles, expression values were normalized per gene by dividing single expression values by the average expression value at all different growth rates. Averages ± standard deviation of these average-normalized values are shown in Figs. 5, 6, 7 and 8.

Gene expression clusters were analysed for overrepresentation of functional annotation categories from the Munich Information Centre for Protein Sequences (MIPS) database (http://mips.gsf.de/genre/proj/yeast), the Gene Ontology (GO) database (http://geneontology.org/) and transcription factor binding (TF) according to [78], based on the hypergeometric distribution analysis tool described by Knijnenburg et al. [79]. Additional functional categories that were searched for enrichment originate from [30, 35, 80, 81] and are listed in Additional file 14.

## Additional files

10.1186/s12934-016-0501-z Set of differentially expressed genes during the transition from exponential growth to stationary phase in aerobic batches of *S. cerevisiae* with glucose as carbon source [34], and overlap with the set of differentially expressed genes during aerobic retentostat cultures in the present study.
10.1186/s12934-016-0501-z Number of genes identified in cluster 1 and 2 that overlap with genes identified in previous growth studies of which the expression correlated with specific growth rate. “Repressed” indicates a positive correlation, “induced” indicates a negative correlation, referring to the mode of regulation at low specific growth rates. Numbers outside the circle indicate the number of genes identified by the corresponding study, but not identified in the present study.
10.1186/s12934-016-0501-z Genes with significant growth-rate dependent gene expression in aerobic and anaerobic cultures between specific growth rates of 0.1 h^-1^ and below 0.001 h^-1^ (see Fig. 6).
10.1186/s12934-016-0501-z Residual glucose concentrations and specific glucose-consumption rate of aerobic and anaerobic cultures during prolonged retentostat. The data is represented as the average ± standard deviation of replicate independent retentostat samples. The residual glucose concentration data point at 0.1 h^-1^ originates from aerobic glucose-limited chemostat cultures described in [82], operated and sampled under the same conditions as reported in the "Methods" section of this work.
10.1186/s12934-016-0501-z Three files encompassing the MATLAB model to predict biomass accumulation, specific growth rate, and specific glucose consumption rates over the course of retentostat (see "Methods" section). Additional file 5 describes how to run or adapt the prediction model. The model itself is contained in two Matlab files (Additional files 6 and 7).
10.1186/s12934-016-0501-z See Additional file 5 caption.

10.1186/s12934-016-0501-z See Additional file 5 caption.
10.1186/s12934-016-0501-z Five files encompassing the MATLAB model for non-linear regression of biomass accumulation in retentostat (see "Methods" section). Additional file 8 describes how to run or adapt the regression model. Additional file 9 is an example Excel file that can be adapted to include (hypothetical) experimental data. In addition, MATLAB writes the output of the regression model to this Excel file. The model itself is contained in three Matlab files (Additional files 10, 11 and 12) which operate together with Additional file 9.
10.1186/s12934-016-0501-z See Additional file 8 caption.
10.1186/s12934-016-0501-z See Additional file 8 caption.
10.1186/s12934-016-0501-z See Additional file 8 caption.
10.1186/s12934-016-0501-z See Additional file 8 caption.
10.1186/s12934-016-0501-z Averaged normalized gene expression of housekeeping genes for *S. cerevisiae* strain CEN.PK113-7D [75]. Dotted bars indicate 20 % variation around normalized expression.
10.1186/s12934-016-0501-z List of gene-groups described in literature that were mined for enrichment among differentially expressed genes identified in clusters 1 and 2 in Fig. 6.

## Abbreviations

µ: specific growth rate
q_P_: specific production rate
q_S_: specific substrate consumption rate
Y_X/S_^max^: maximum biomass yield on substrate
m_S_: specific rate of substrate consumption for maintenance
m_ATP_: specific rate of ATP consumption for maintenance
C_S_: growth-limiting substrate (glucose) concentration
C_S,MC_: glucose concentration in medium reservoir during chemostat phase
C_S,MR_: glucose concentration in medium reservoir during retentostat phase
V_S_: volume mixing vessel
ɸ_V_: flow rate
