# Supplementary figures and images for: Maintenance-energy requirements and robustness of Saccharomyces cerevisiae at aerobic near-zero specific growth rates
Source: Microb Cell Fact. 2016 Jun 17;15:111. doi: 10.1186/s12934-016-0501-z (PMC4912818; doi:10.1186/s12934-016-0501-z)

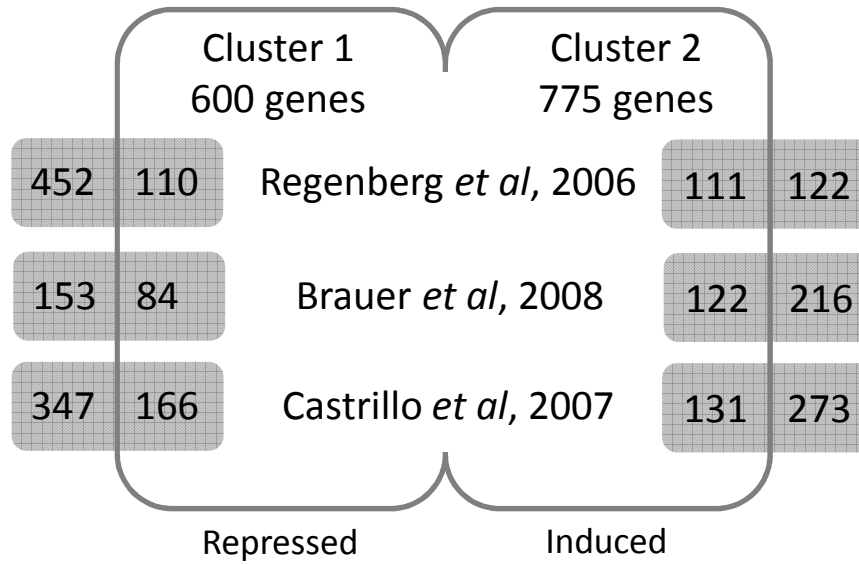

Supplement: Supplementary file 2 — 10.1186/s12934-016-0501-z Number of genes identified in cluster 1 and 2 that overlap with genes identified in previous growth studies of which the expression correlated with specific growth rate. “Repressed” indicates a positive correlation, “induced” indicates a negative correlation, referring to the mode of regulation at low specific growth rates. Numbers outside the circle indicate the number of genes identified by the corresponding study, but not identified in the present study. [file 12934_2016_501_MOESM2_ESM.pdf]

(a)

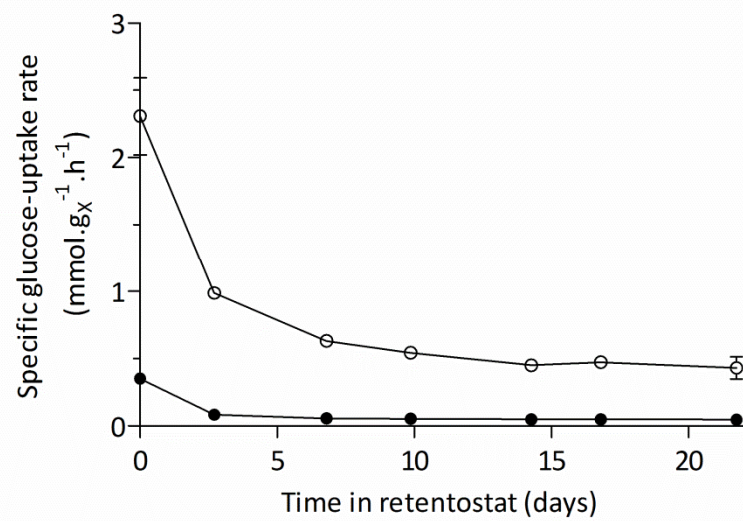

(b)

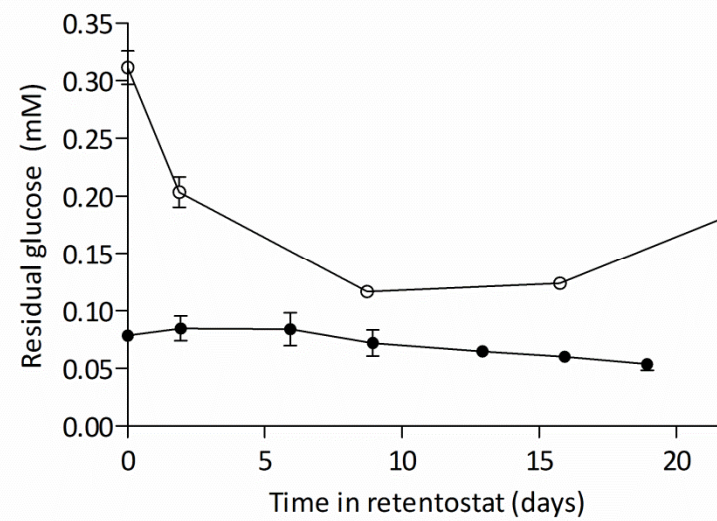

Supplement: Supplementary file 4 — 10.1186/s12934-016-0501-z Residual glucose concentrations and specific glucose-consumption rate of aerobic and anaerobic cultures during prolonged retentostat. The data is represented as the average ± standard deviation of replicate independent retentostat samples. The residual glucose concentration data point at 0.1 h-1 originates from aerobic glucose-limited chemostat cultures described in [82], operated and sampled under the same conditions as reported in the "Methods" section of this work. [file 12934_2016_501_MOESM4_ESM.pdf]

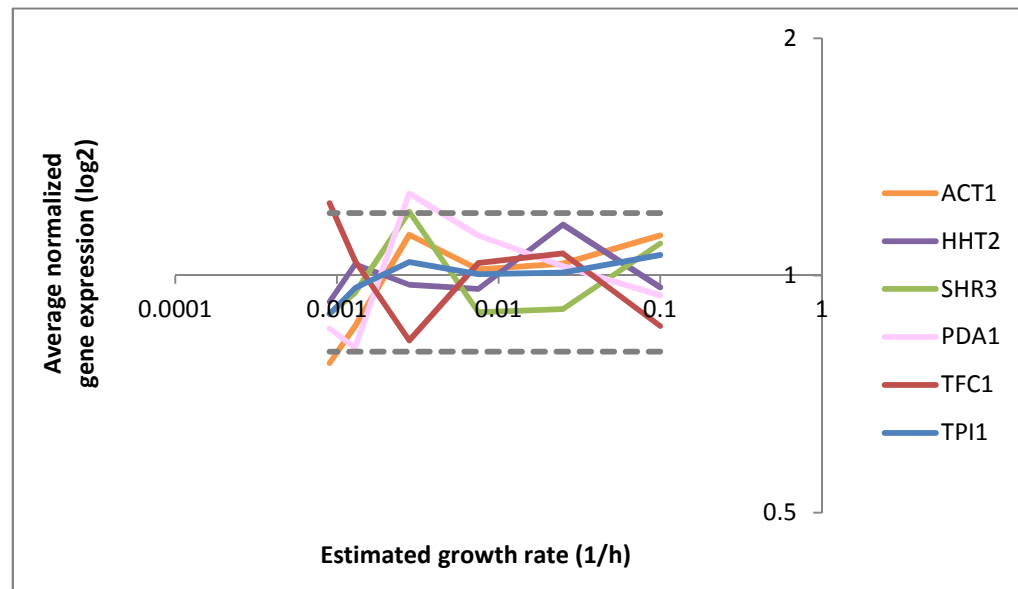

Supplement: Supplementary file 13 — 10.1186/s12934-016-0501-z Averaged normalized gene expression of housekeeping genes for S. cerevisiae strain CEN.PK113-7D [75]. Dotted bars indicate 20 % variation around normalized expression. [file 12934_2016_501_MOESM13_ESM.pdf]
