# Supplementary material for: Maintenance-energy requirements and robustness of Saccharomyces cerevisiae at aerobic near-zero specific growth rates
Source: Microb Cell Fact. 2016 Jun 17;15:111. doi: 10.1186/s12934-016-0501-z (PMC4912818; doi:10.1186/s12934-016-0501-z)
Supplement: Supplementary file 5 — 10.1186/s12934-016-0501-z Three files encompassing the MATLAB model to predict biomass accumulation, specific growth rate, and specific glucose consumption rates over the course of retentostat (see "Methods" section). Additional file 5 describes how to run or adapt the prediction model. The model itself is contained in two Matlab files (Additional files 6 and 7). [file 12934_2016_501_MOESM5_ESM.pdf]

HOW TO – Matlab prediction model

Guide to supplementary data accompanying the manuscript by Vos et al. 2016

Apart from this HOW TO document, Additional file 6 (a,b,c) encompasses two Matlab files (.m). Together, these documents allow to do a prediction analysis for biomass accumulation and biomass specific rates of substrate consumption and growth in retentostat cultures. Files have to be kept in the same folder to function appropriately in MATLAB.

File *Saccharomyces_cerevisiae.m* is the MATLAB file that has to be run in MATLAB to do a prediction analysis. When executed, it calls for the function in the file *Retentostat_growth.m*. The function in the latter file contain the ordinary differential equations (ODEs) that are needed to describe biomass accumulation and corresponding rates of growth and substrate consumption in the retentostat (see Methods section Vos *et al.* 2016). These ODEs are solved multiple times for varying values of ms (maintenance energy requirements m_S), and V_A (mixing vessel).

Data are plotted in MATLAB and presented as three dimensional plots depicting on the different axis, biomass concentration, the maintenance coefficient, and the volume of the mixing vessel. A final plot depicts the growth kinetics in retentostat for a minimum and maximum value of mS and a chosen working volume in the mixing vessel described in the command window after the prediction is finished, shown below in red:

```
"The maximal volume of mixing vessel A for which a robust set-up can be guaranteed based on this model, up to an ms of 0.012 is a volume of 1.3 L."
```

To personalize the files for prediction of new retentostat experiments with other organisms or different conditions, one can change the values in file *Saccharomyces_cerevisiae.m*:

```
V_B = 1.4; % L (Bioreactor working volume)
Cs_chem = 20 ; % g/L    (glucose concentration in medium reservoir during chemostat)
Cs_ret = 7.5 ; % g/L    (glucose concentration in medium reservoir during retentostat)
Ysx_max = 0.5; % g/g    (maximum biomass yield on glucose)
Ysp_max = 0.01; % g/g   (maximum product yield on glucose) Must be a non-zero value!
ms_est = 0.011; % gs/gx/h     (initial estimate of the maintenance coefficient)
D_chem = 0.025; % h-1   (dilution rate)
F_in = V_B*D_chem; % L/h      (medium flow rate)
days = 25; % d                (retentostat operation time)
```

Optional product terms can be included as well: for linear qp (mu)-relations (qp = a*mu+b) qp_func = parameter a and qp_res = parameter b, with qp [mol·g_X^{-1}·h^{-1}] and Ysp_max in [mol_P·mol_S^{-1}]. For non-producing strains qp terms equal 0:

```
% for linear qp(mu)-relations qp = a*mu+b
qp_func = 0; % parameter a
qp_res = 0; % parameter b
```

In file *Retentostat_growth.m* (Lines 21-25), values for volume of the mixing vessel (V_A) described according to:

```
for p = 1:maxp
    %% Process Parameters
    % Vessel A - Mixing Vessel
    %     Fin_A = 0.035; % L/h
    V_A = 0 + p*0.1; %L
```

and range between 0 and 2 L.

In File *Retentostat_growth.m* (Lines 69), values for the maintenance requirements (ms) described according to:

```
ms = (ms_est-maxi/2*0.0001) + i*0.0001 % maintenance coefficient % gs/gx/h
```

and range between +10% and -10% of ms_est in file *Saccharomyces_cerevisiae.m*.
