# Supplementary material for: Maintenance-energy requirements and robustness of Saccharomyces cerevisiae at aerobic near-zero specific growth rates
Source: Microb Cell Fact. 2016 Jun 17;15:111. doi: 10.1186/s12934-016-0501-z (PMC4912818; doi:10.1186/s12934-016-0501-z)
Supplement: Supplementary file 8 — 10.1186/s12934-016-0501-z Five files encompassing the MATLAB model for non-linear regression of biomass accumulation in retentostat (see "Methods" section). Additional file 8 describes how to run or adapt the regression model. Additional file 9 is an example Excel file that can be adapted to include (hypothetical) experimental data. In addition, MATLAB writes the output of the regression model to this Excel file. The model itself is contained in three Matlab files (Additional files 10, 11 and 12) which operate together with Additional file 9. [file 12934_2016_501_MOESM8_ESM.pdf]

HOW TO – Matlab regression model

Guide to supplementary data accompanying the manuscript by Vos et al. 2016

Apart from this HOW TO document, Additional file 7 (a,b,c,d,e) encompasses three Matlab files (.m) and one Excel file (.xlsx). Together, these documents allow to do a regression analysis on experimental data from retentostat cultures, and have to be kept in the same folder to function appropriately.

Excel sheet *MATLABread* in the file *Example_retentostat_data.xlsx*, contains data collected from an example retentostat experiment. In here, Cx refers to the biomass concentration, DW refers to the measured dry-weight (see Methods section Vos *et al.* 2016), and Cs_in refers to the glucose concentration in the feed. The different volumes and flow rates taken into account by the model are indicated in the data sheet as well.

File *Example_rententostat_data.m* is the MATLAB file that has to be run in MATLAB to do a regression analysis. When executed, it automatically requests data from Sheet *MATLABread* in the file *Example_retentostat_data.xlsx*, and calls for the function in the file *Retentostat_regression_Sc_kd.m*. The function in the latter file contain the ordinary differential equations (ODEs) that are needed to describe (viable and total) biomass accumulation in the retentostat (see Methods section Vos *et al.* 2016), which are solved multiple times for varying values of ms (maintenance energy requirements m_S), and kd (death rate k_d) until the sum of square errors (SSE) is minimized.

Data is plotted using the file *retentostat_plotting.m*.

Sheet *Modelling Summary* in the Excel file *Example_retentostat_data.xlsx* is where MATLAB deposits the data after a complete regression analysis. In here, ms opt is the value for m_S (in $mg \cdot g^{-1} \cdot h^{-1}$) corresponding to the regression with the lowest SSE. In addition, values for SSE, r-squared (Rsq), optimised death rate (Kd [“value”$\cdot 10^{-3}$ h^{-1}]) and the final specific growth rate at the end of retentostat (in h^{-1}). Furthermore, the glucose concentration in the feed (Cs), the biomass concentration in the retentostat (Cx) the specific growth rate (mu) and the specific substrate uptake rate (qs) are published according to the regression analysis, with a step size of 30 minutes.

To personalize the files for new retentostat experiments with other organisms or different conditions, one can change the following values, arranged according to the files they appear in:

File *Example_rententostat_data.xlsx*

- Change all data in sheet *MATLABread* according to your personal experimental data
- Sheet *Modelling Summary* is automatically emptied and filled with each new regression analysis.

File *Example_rententostat_data.m*

- Line 7-11, yeast parameters (qp and Ysp_max refers to production characteristics of a strain*)
- Line 13-23, the data location that is read by MATLAB from an Excel file.

File *Retentostat_regression_Sc_kd.m*

- Line 18-19, initial guesses for ms and kd (in $g_{glucose} \cdot g_{biomass}^{-1} \cdot h^{-1}$ and in h^{-1}, respectively), starting point for solving ODEs and minimizing SSE.
- Line 33, time span of retentostat (in days)
- Line 214, concentration in the medium reservoir during retentostat

* Optional product terms: for linear qp (mu)-relations (qp = a*mu+b), qp_func = parameter a and qp_res = parameter b, with qp [$g \cdot g_X^{-1} \cdot h^{-1}$] and Ysp_max in [$g_P \cdot g_S^{-1}$]. For non-producing strains qp terms equal 0.
